# Diversification processes of teleost intron-less opsin genes

**DOI:** 10.1016/j.jbc.2023.104899

**Published:** 2023-06-07

**Authors:** Chihiro Fujiyabu, Keita Sato, Hideyo Ohuchi, Takahiro Yamashita

**Affiliations:** 1 Department of Biophysics, Graduate School of Science, Kyoto University, Kyoto, Japan; 2 Department of Cytology and Histology, Okayama University Faculty of Medicine, Dentistry and Pharmaceutical Sciences, Okayama, Japan

**Keywords:** rhodopsin, melanopsin, G protein-coupled receptor (GPCR), retrogene, molecular evolution

## Abstract

Opsins are universal photosensitive proteins in animals. Vertebrates have a variety of opsin genes for visual and non-visual photoreceptions. Analysis of the gene structures shows that most opsin genes have introns in their coding regions. However, teleosts exceptionally have several intron-less opsin genes that are presumed to have been duplicated by an RNA-based gene duplication mechanism, retroduplication. Among these retrogenes, we focused on the *Opn4* (melanopsin) gene responsible for non-image-forming photoreception. Many teleosts have five *Opn4* genes including one intron-less gene, which is speculated to have been formed from a parental intron-containing gene in the Actinopterygii. In this study, to reveal the evolutionary history of *Opn4* genes, we analyzed them in teleost (zebrafish and medaka) and non-teleost (bichir, sturgeon, and gar) fishes. Our synteny analysis suggests that the intron-less *Opn4* gene emerged by retroduplication after the branching of the bichir lineage. In addition, our biochemical and histochemical analyses showed that, in the teleost lineage, the newly acquired intron-less *Opn4* gene became abundantly used without substantial changes in the molecular properties of the Opn4 protein. This stepwise evolutionary model of *Opn4* genes is quite similar to that of *rhodopsin* genes in the Actinopterygii. The unique acquisition of *rhodopsin* and *Opn4* retrogenes would have contributed to the diversification of the opsin gene repertoires in the Actinopterygii and the adaptation of teleosts to various aquatic environments.

Vertebrates utilize light not only for visual functions but also for various non-visual functions. These photoreceptions are generally underlain by photoreceptive proteins, opsins (1, 2, 3). Recent advances in genome analysis have revealed that most vertebrates have multiple opsin genes in their genomes. Opsins share amino acid sequence homology and structural elements including seven transmembrane domains and a chromophore, retinal. Thus, opsin genes are considered to have expanded from a single ancestral gene through gene duplications and mutations, which has led to the diversification of the molecular properties and expression patterns of the proteins that they encode. Opsins can be classified into several groups by phylogenetic relationship analysis. Among mammalian opsins, rhodopsin and Opn4 (melanopsin) are the best-studied opsins responsible for visual and non-visual photoreceptions, respectively. Rhodopsin functions for twilight vision in the rod photoreceptor cells of the retina and belongs to the c-opsin group together with cone visual pigments and Opn3. Rhodopsin binds 11-*cis* retinal in the dark and activates Gt-type of G protein in a light-dependent manner. By contrast, Opn4 functions for several non-image-forming photoreceptions, such as entrainment of the circadian rhythm and pupillary light reflex, in the intrinsically photosensitive retinal ganglion cells of the retina and belongs to the r-opsin group (4, 5). Opn4 binds 11-*cis* retinal in the dark and mainly activates Gq-type of G protein in a light-dependent manner.

Analysis of the gene structures of vertebrate opsins has revealed that most vertebrate opsin genes contain introns in their translational regions and several boundary positions of exons and introns are conserved within each opsin group (6). These facts indicate that opsin genes have been diversified mainly by DNA-based gene duplication mechanisms, such as whole-genome duplication, unequal crossing over, or DNA transposons (7, 8). However, it has been reported that a few opsin genes do not contain introns in their coding regions (9, 10, 11). These intron-less (single-exon) genes are supposed to have been acquired by the RNA-based gene duplication mechanism, namely, retroduplication (retrotransposition), in which cDNA was reverse-transcribed from mature mRNA of a parental intron-containing gene and was inserted into another region of the genome (12). In vertebrates, several intron-less opsin genes have been found in fish genomes. In a previous study, we analyzed the evolutionary process of the teleost intron-less *rhodopsin* gene (13). In general, vertebrates have a single intron-containing *rhodopsin* gene in their genomes and utilize it in rod cells for visual photoreception. By contrast, teleosts have intron-containing and intron-less *rhodopsin* genes in their genomes. The intron-less (*Rh1*) and intron-containing (*Exorh*) genes are expressed in the rod cells (9) and the pineal gland (14), respectively. Taking account of the phylogenetic relationship of actinopterygian species (ray-finned fishes) (15), we analyzed the *rhodopsin* genes of non-teleost fishes in the Actinopterygii (Polypteriformes, Acipenseriformes, and Holostei). Our analysis led us to propose an evolutionary scenario in which the intron-less *rhodopsin* gene emerged from a parental intron-containing one by retroduplication after branching of the Polypteriformes and the intron-containing gene changed its expression pattern to exclusive and abundant distribution in the pineal gland after branching of the Holostei.

Another well-known example of teleost intron-less opsin genes is *Opn4*. The *Opn4* gene was first discovered in the melanophores of *Xenopus laevis* (16) and was subsequently identified also in the human genome (17). Accumulated information about *Opn4* genes has shown that vertebrate *Opn4* genes are classified into two lineages, *Opn4m* (mammalian-like) and *Opn4x* (*Xenopus*-like), in the phylogenetic tree and mammals have a single *Opn4* gene (*Opn4m*) due to loss of the *Opn4x* gene (18). Previous reports about Opn4 in non-mammalian vertebrates showed that Opn4 protein is expressed in several tissues, including the retina, brain, and skin (18), suggesting the possibility that Opn4 protein contributes to various non-visual photoreceptions (4). The genomes of many teleost fishes contain five *Opn4* genes, *Opn4m1*, *Opn4m2*, *Opn4m3*, *Opn4x1*, and *Opn4x2*, among which *Opn4m2* is an intron-less single-exon gene (19). A previous study revealed that zebrafish *Opn4* genes show largely non-overlapping expression patterns in the retina and the intron-less *Opn4m* gene (*Opn4m2*) is predominantly expressed in the inner nuclear layer (INL) and the photoreceptor cells (19, 20). Thus, it has been supposed that, during the evolutionary process of the Actinopterygii, the *Opn4* genes expanded by both retroduplication and teleost-specific whole-genome duplication and functionally differentiated by the diversification of their expression patterns.

In this study, we analyzed the synteny conservation of the *Opn4* genes in addition to the *rhodopsin* genes among actinopterygian species. We also examined the molecular properties of Opn4 protein and the tissue distributions of *Opn4* mRNA in actinopterygian species. Our comparison of the diversification processes of intron-containing and intron-less *rhodopsin* and *Opn4* genes suggests a common evolutionary history of these two opsin genes in the Actinopterygii.

## Results

### Synteny analysis of *rhodopsin* genes in the Actinopterygii

**Figure 1:**
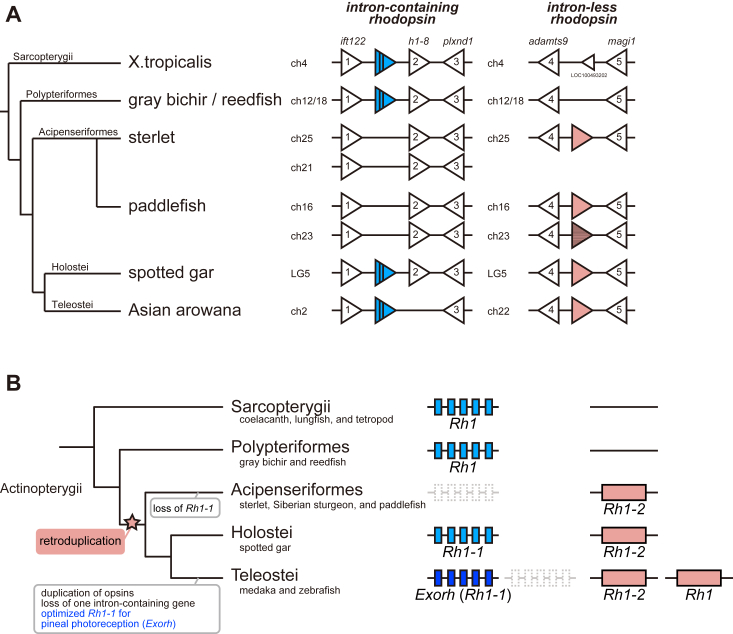
**Comparison of *rhodopsin* gene repertoires in the Actinopterygii**. *A*, synteny of orthologous genes flanking the intron-containing *rhodopsin* gene (*blue triangle* with *vertical lines*) and the intron-less *rhodopsin* gene (*pink triangle*) in actinopterygian species and *Xenopus tropicalis*. One of the two intron-less *rhodopsin* genes in paddlefish is a pseudogene (*pink triangle* with *horizontal stripes*) which contains two stop codons in the coding region. The genes flanking the *rhodopsin* loci are shown by white triangles. Gene names are indicated above the *X. tropicalis* genes, intraflagellar transport 122 homolog (*ift122*) (gene number 1 shown by *white triangle*), H1.8 linker histone (*h1-8*) (gene number 2), plexin D1 (*plxnd1*) (gene number 3), ADAM metallopeptidase with thrombospondin type 1 motif 9 (*adamts9*) (gene number 4) and membrane-associated guanylate kinase, WW and PDZ domain containing 1 (*magi1*) (gene number 5). The animals of the NCBI assembly accession number: *X. tropicalis*, GCF_000004195.4; gray bichir (*P. senegalus*), GCF_016835505.1; reedfish (*E. calabaricus*), GCF_900747795.1; sterlet (*A. ruthenus*), GCF_010645085.1; paddlefish (*P. spathul*), GCF_017654505.1; spotted gar (*L. oculatus*), GCF_000242695.1; Asian arowana (*S. formosus*), GCF_900964775.1. Detailed gene information is shown in Data S1. *B*, evolutionary model of teleost intron-containing and intron-less *rhodopsin* genes in the Actinopterygii. Fishes in the Polypteriformes have only one *rhodopsin* gene (*Rh1*), which contains introns. After branching of the Polypteriformes, the intron-less *rhodopsin* gene (*Rh1-2*) emerged by retroduplication. The intron-containing *rhodopsin* gene (*Rh1-1*) was lost in the Acipenseriformes. The ancestor of the Teleostei duplicated the intron-less *rhodopsin* gene (*Rh1-2*, *Rh1*) by a whole genome duplication and changed the expression pattern of the intron-containing *rhodopsin* gene (*Exorh* (*Rh1-1*)) for pineal photoreception (13). The nomenclature of *rhodopsin* genes we use refers to that in the previous studies (36, 49, 50, 51).

In our previous study, we obtained the *rhodopsin* clones from several non-teleost fishes in the Actinopterygii and proposed a stepwise evolutionary model of teleost intron-containing and intron-less *rhodopsin* genes, that is, the intron-less *rhodopsin* gene was retroduplicated from an ancestral intron-containing *rhodopsin* gene after the branching of the Polypteriformes and subsequently the molecular property and tissue distribution of the ancestral intron-containing *rhodopsin* gene changed to be exclusively utilized for pineal photoreception in teleosts (13). After our analysis, the whole genome assemblies of gray bichir (*Polypterus senegalus*) and reedfish (*Erpetoichthys calabaricus*) in the Polypteriformes and sterlet (*Acipenser ruthenus*) and paddlefish (*Polyodon spathula*) in the Acipenseriformes were published in the public databases (21, 22). To evaluate our previous model, we examined the genomic locations of these *rhodopsin* genes and compared the syntenies flanking the *rhodopsin* genes in the genomes of actinopterygian species based on Actinopterygii evolution (15) (Fig. 1*A* and Data S1). The genomes of the Acipenseriformes (sterlet and paddlefish), the Holostei (spotted gar (*Lepisosteus oculatus*)), and the Teleostei (Asian arowana (*Scleropages formosus*)) contain the intron-less *rhodopsin* gene in the conserved synteny, whereas the genomes of the Polypteriformes (gray bichir and reedfish) do not contain the *rhodopsin* gene in the corresponding synteny. These results suggest that an intron-less *rhodopsin* gene emerged by retroduplication of the parental intron-containing *rhodopsin* gene and was inserted into the genomic location between *adamts9* and *magi1* after branching of the Polypteriformes. In addition, the intron-containing *rhodopsin* gene is found in the conserved synteny of the Asian arowana, spotted gar, gray bichir, and reedfish genomes but is missing in the sterlet and paddlefish genomes. This is consistent with our previous analysis in which we could not obtain the clone of an intron-containing *rhodopsin* gene in Siberian sturgeon. These findings suggested that the intron-containing gene was lost at the base of the Acipenseriformes. It should be noted that, in the Acipenseriformes, sterlet has one intron-less *rhodopsin* gene and paddlefish has two intron-less *rhodopsin* genes, a protein-coding gene and a pseudogene, which emerged by the whole genome duplication. Thus, both species have only one intron-less *rhodopsin* gene. These synteny analyses supported our evolutionary model of *rhodopsin* genes in the Actinopterygii (Fig. 1*B*).

### Synteny analysis of *Opn4* genes in the Actinopterygii

**Figure 2:**
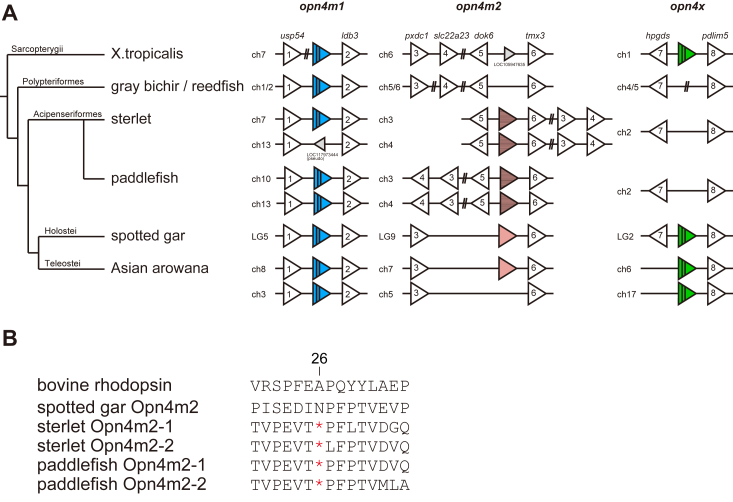
**Comparison of *Opn4* gene repertoires in the Actinopterygii**. *A*, synteny of orthologous genes flanking the *Opn4m1* gene (*blue triangle* with *vertical lines*), *Opn4m2* gene (*pink triangle*), and *Opn4x* gene (*green triangle* with *vertical lines*) in actinopterygian species and *X. tropicalis*. The intron-less *Opn4m2* genes in the Acipenseriformes are pseudogenes (*pink triangle* with *horizontal stripes*) which contain a stop codon at the same position in their coding regions (*B*). The genes flanking the *Opn4* loci are shown by *white triangles*. Gene names are indicated above the *X. tropicalis* genes, ubiquitin specific peptidase 54 (*usp54*) (gene number 1 shown by white triangle), LIM domain binding 3 (*ldb3*) (gene number 2), PX domain containing 1 (*pxdc1*) (gene number 3), solute carrier family 22 member 23 (*slc22a23*) (gene number 4), docking protein 6 (*dok6*) (gene number 5), thioredoxin related transmembrane protein 3 (*tmx3*) (gene number 6), hematopoietic prostaglandin D synthase (*hpgds*) (gene number 7) and PDZ, and LIM domain 5 (*pdlim5*) (gene number 8). The animals of the NCBI assembly accession number: *X. tropicalis*, GCF_000004195.4; gray bichir (*P. senegalus*), GCF_016835505.1; reedfish (*E. calabaricus*), GCF_900747795.1; sterlet (*A. ruthenus*), GCF_010645085.1; paddlefish (*P. spathul*), GCF_017654505.1; spotted gar (*L. oculatus*), GCF_000242695.1; Asian arowana (*S. formosus*), GCF_900964775.1. Detailed gene information is shown in Data S1. *B*, comparison of deduced amino acid residues between those encoded by the intron-less *Opn4m2* gene of spotted gar and the intron-less *Opn4m2* pseudogenes of sterlet and paddlefish. The sequences of Opn4m2 are aligned with the sequence of bovine rhodopsin (BAB83621.1). Both sterlet and paddlefish have two intron-less *Opn4m2* pseudogenes (*Opn4m2-1* and *Opn4m2-2*), all of which share a nonsense mutation site at position 26 (based on the bovine rhodopsin numbering system). Gene IDs of these sequences are as follows: spotted gar *Opn4m2*, 102695598; sterlet *Opn4m2-1*, 117400101; sterlet *Opn4m2-2*, 117394593; paddlefish *Opn4m2-1*, 121307772. We found the sequence of paddlefish *Opn4m2-2* using blast search in the whole-genome shotgun sequence, JADDYA010000170.1 (127199–127727).

Next, we compared the repertoires of *Opn4* genes among actinopterygian species. The *Xenopus tropicalis* genome contains two intron-containing *Opn4* genes (*Opn4m* and *Opn4x*) and the Asian arowana genome contains four intro-containing *Opn4* genes (*Opn4m1*, *Opn4m3*, *Opn4x1*, and *Opn4x2*) and one intron-less *Opn4* gene (*Opn4m2*). Thus, we searched for intron-containing and intron-less *Opn4* genes in the genomes of non-teleost fishes in the Actinopterygii (Fig. 2*A* and Data S1). The spotted gar genome contains an *Opn4m2* protein-coding gene and the sterlet and paddlefish genomes contain two *Opn4m2* pseudogenes (Fig. 2*B*). These intron-less *Opn4m* genes are located upstream of *tmx3* in the genomes. By contrast, *Opn4m2* gene is missing in the corresponding synteny of the gray bichir and reedfish genomes. Thus, we speculate that the intron-less *Opn4m* gene emerged by retroduplication of the parental intron-containing *Opn4m* gene (*Opn4m*) after the branching of the Polypteriformes. The parental intron-containing *Opn4m* gene is located in the conserved synteny, between *usp54* and *ldb3*, of the Asian arowana, spotted gar, sterlet, paddlefish, gray bichir, and reedfish genomes, among which the Asian arowana and paddlefish genomes have two intron-containing *Opn4m* genes because of the whole genome duplication. The other intron-containing *Opn4* gene, *Opn4x*, is identified in the spotted gar genome. The location of *Opn4x* gene in the genome is conserved in upstream of *pdlim5* among Asian arowana, spotted gar, and *X. tropicalis*. By contrast, *Opn4x* gene is missing in the corresponding synteny of the sterlet, paddlefish, gray bichir, and reedfish genomes. This suggests that *Opn4x* gene was independently lost in the Polypteriformes and Acipenseriformes lineages. Therefore, these synteny analyses showed that the repertoires of *Opn4* genes in non-teleost fishes have been diversified by retroduplication, whole genome duplication, and gene loss including pseudogenization.

### Analysis of the molecular properties of Opn4 proteins

Next, we analyzed the molecular properties of Opn4 proteins among actinopterygian species. We obtained full-length *Opn4* cDNAs from gray bichir in the Polypteriformes (*Opn4m*), Siberian sturgeon (*Acipenser baerii*) in the Acipenseriformes (*Opn4m1*), spotted gar in the Holostei (*Opn4m1*, *Opn4m2*, and *Opn4x*), and medaka in the Teleostei (*Opn4m1*, *Opn4m2*, *Opn4m3*, *Opn4x1*, and *Opn4x2*). To improve the expression level of Opn4 proteins, we truncated the C-terminus according to our previous study on mouse Opn4 (23) and added the Rho1D4 epitope sequence as a purification tag. It has been reported that the truncation of the C-terminus does not affect the photoreaction of cephalopod rhodopsins, which are closely related to vertebrate Opn4 in the phylogenetic tree of opsins (24, 25). In addition, it should be noted that the photochemical properties of the C-terminal truncated protein of mouse Opn4 (23) are consistent with the spectral sensitivities of Opn4-expressing ganglion cells that were measured by the electrophysiological recording in the mouse retina (26).

**Figure 3:**
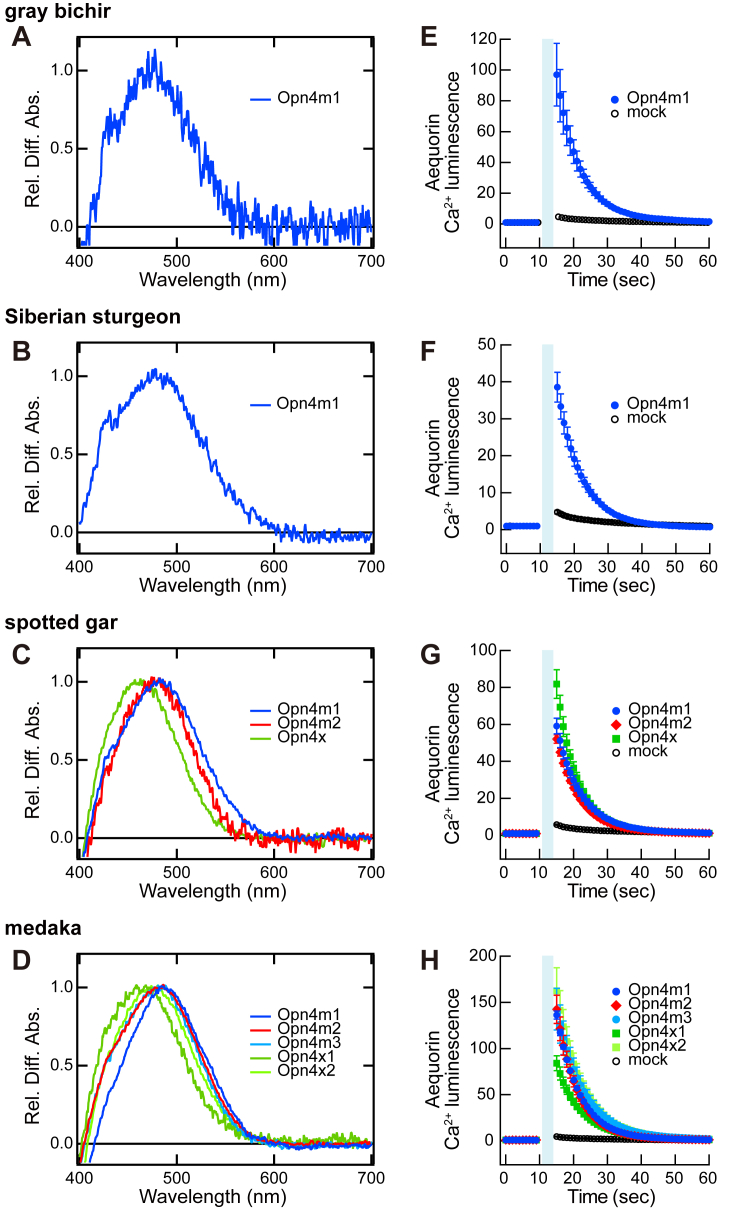
**Molecular characteristics of Opn4 proteins**. *A* and *B*, difference absorption spectra of gray bichir Opn4m protein (*A*) and Siberian sturgeon Opn4m1 protein (*B*). The Opn4-expressing cell membranes were mixed with 11-*cis* retinal and solubilized with 1% DDM. The absorption spectra of the extracts were measured before and after *yellow light* (>500 nm) irradiation in the presence of 30 mM hydroxylamine. Difference spectra were calculated by subtracting the spectrum after light irradiation from that before irradiation. λmax of gray bichir Opn4m protein and Siberian sturgeon Opn4m1 protein were estimated to be at 473 nm and 479 nm, respectively. *C*, difference absorption spectra of spotted gar Opn4m1 protein (*blue*), Opn4m2 protein (*red*) and Opn4x protein (*green*). λmax of Opn4m1, Opn4m2 and Opn4x protein were estimated to be 482 nm, 477 nm, and 461 nm, respectively. *D*, difference absorption spectra of medaka Opn4m1 protein (*blue*), Opn4m2 protein (*red*), Opn4m3 protein (*light blue*), Opn4x1 protein (*green*) and Opn4x2 protein (*light green*). λmax of Opn4m1, Opn4m2, Opn4m3, Opn4x1 and Opn4x2 protein were estimated to be 487 nm, 482 nm, 480 nm, 463 nm, and 474 nm, respectively. *E–H*, light-induced changes of intracellular Ca^2+^ level by Opn4 protein. The Ca^2+^ level in the HEK293S cells expressing gray bichir Opn4m protein (*E*), Siberian sturgeon Opn4m1 protein (*F*), and spotted gar Opn4 protein (Opn4m1, Opn4m2 or Opn4x) (*G*) or medaka Opn4 protein (Opn4m1, Opn4m2, Opn4m3, Opn4x1 or Opn4x2) (*H*) was measured using aequorin-based luminescent assay. Ca^2+^-dependent luminescence change of aequorin was triggered by light irradiation from blue (450 nm) LED for 5 s. Data are presented as the means ± deviations of two independent experiments compared with those of mock-transfected cells.

We expressed the recombinant Opn4 proteins in cultured cells and reconstituted the corresponding photo-pigments (Fig. 3, *A*–*D*). To measure the absorption maximum (λmax) of these Opn4 proteins, we added 11-*cis* retinal to the Opn4 protein-containing cell membranes and solubilized them with 1% dodecyl maltoside (DDM). We irradiated the extracts with yellow light (>500 nm) in the presence of 30 mM hydroxylamine and calculated their difference spectra by subtracting the spectrum after light irradiation from that before irradiation. Based on these spectra, we estimated λmax of gray bichir Opn4m (473 nm) and Siberian sturgeon Opn4m1 (479 nm) proteins (Fig. 3, *A* and *B*). In addition, the estimated absorption maximum (λmax) of spotted gar Opn4m1 (482 nm) and Opn4m2 (477 nm) proteins were red-shifted compared to that of spotted gar Opn4x protein (461 nm) (Fig. 3*C*). Likewise, λmax of medaka Opn4m1 (487 nm), Opn4m2 (482 nm), and Opn4m3 (480 nm) proteins were red-shifted compared to those of medaka Opn4x1 (463 nm) and Opn4x2 (474 nm) proteins (Fig. 3*D*). We also conducted affinity column chromatography to remove other extract components from DDM-solubilized samples of spotted gar Opn4 and medaka Opn4 proteins and obtained their absorption spectra (Fig. S1, *A* and *B*), whose spectral peaks were comparable to the values estimated from Figure 3, *C* and *D*. However, we could not obtain the absorption spectrum of gray bichir Opn4m or Siberian sturgeon Opn4m1 protein after the purification procedure, probably because of their low expression levels in mammalian cultured cells. Taken together, our analysis showed that λmax values of Opn4 proteins from actinopterygian species have a common feature, that is, λmax of Opn4m protein (470–480 nm) is red-shifted compared to that of Opn4x protein (450–470 nm). This is consistent with λmax of Opn4 proteins from other vertebrate species previously reported (19, 23, 27, 28).

We also analyzed the ability of Opn4 proteins from actinopterygian species to activate G protein. Opn4 protein generally activates Gq-type of G protein to increase the intracellular Ca^2+^ concentration in a light-dependent manner. Thus, we measured the change in the intracellular Ca^2+^ level based on aequorin bioluminescence. We expressed the C-terminal truncated gray bichir, Siberian sturgeon, spotted gar, or medaka Opn4 protein with the Rho1D4 epitope sequence in cultured cells and observed an increase in the luminescence from aequorin induced by blue light irradiation (Fig. 3, *E*–*H*), indicating that blue light irradiation can induce the activation of Gq-type of G protein by these Opn4 proteins. We also prepared two constructs, one of which has the full-length sequence with the Rho1D4 epitope sequence and the other of which has the truncated C-terminus without the Rho1D4 epitope sequence, of spotted gar Opn4m1, Opn4m2, and Opn4x. Our analysis confirmed that these two constructs showed a light-dependent elevation in the Ca^2+^ level comparably to the construct which has the truncated C-terminus with the Rho1D4 epitope sequence (Fig. S1, *C*–*E*). Our data showed that these Opn4 proteins can trigger the Ca^2+^ signaling pathway in the cells, although it needs to be confirmed experimentally by using intact cells. Therefore, we concluded that Opn4 proteins encoded by both intron-containing and intron-less *Opn4* genes from actinopterygian species share common spectral and biochemical properties.

### Analysis of the expression patterns of *Opn4* mRNA in the retina

**Figure 4:**
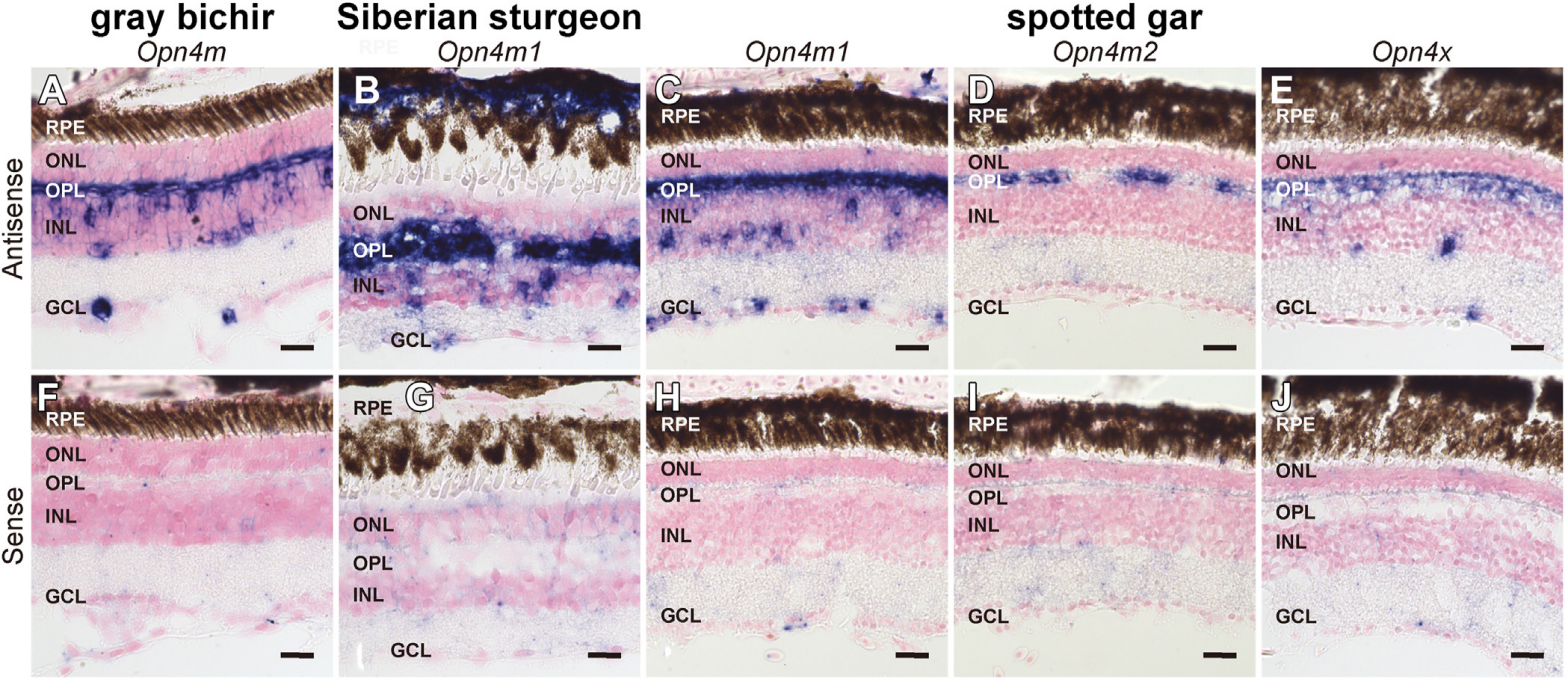
**Distribution of *Opn4* mRNA in the retina of non-teleost fishes in the Actinopterygii.** Distribution of the transcripts of gray bichir *Opn4m* (*A* and *F*), Siberian sturgeon *Opn4m1* (*B* and *G*) and spotted gar *Opn4m1* (*C* and *H*), *Opn4m2* (*D* and *I*) and *Opn4x* (*E* and *J*) in the retina. These sections were hybridized with antisense probes (*A*–*E*) or corresponding sense probes (*F*–*J*). Scale bar: 20 μm.

To elucidate whether *Opn4* genes changed their mRNA distribution patterns during the evolutionary process in the Actinopterygii, we conducted *in situ* hybridization analysis in the retinas of non-teleost fishes: gray bichir, Siberian sturgeon, and spotted gar. Our analysis showed that the gray bichir intron-containing *Opn4* gene (*Opn4m*) was expressed abundantly in the cells (presumed horizontal cells) abutting the outer plexiform layer (OPL) (Fig. 4, *A* and *F*). We also detected gray bichir *Opn4m* mRNA in a small number of the presumed amacrine cells and bipolar cells or Müller cells on the INL and retinal ganglion cells. Likewise, the Siberian sturgeon intron-containing *Opn4* gene (*Opn4m1*) showed high expression in the cells abutting the OPL and a small number of the cells on the INL and ganglion cell layer (GCL) (Fig. 4, *B* and *G*). Additionally, Siberian sturgeon *Opn4m1* mRNA was detected in the retinal pigment epithelium (RPE) cells. Next, we analyzed the expression patterns of the spotted gar *Opn4* genes. The transcript of the spotted gar intron-containing *Opn4* gene (*Opn4m1*) was distributed abundantly in the presumed horizontal cells and in a subset of cells in the INL and GCL, which covered the distribution areas of gray bichir *Opn4m* and Siberian sturgeon *Opn4m1* mRNAs (Fig. 4, *C* and *H*). In addition, we could detect spotted gar *Opn4m1* mRNA signals in the RPE cells in the fundus of the retina (Fig. S2). On the other hand, the hybridization signals of mRNA of the spotted gar intron-less *Opn4m* gene (*Opn4m2*) were restricted to a quite small number of the cells abutting the OPL (Fig. 4, *D* and *I*). The spotted gar *Opn4x* gene (*Opn4x*) was expressed weakly in the OPL and in a sparse population of the cells in the ONL and GCL, which is similar to the expression patterns of spotted gar *Opn4m* gene (*Opn4m1*) (Fig. 4, *E* and *J*).

**Figure 5:**
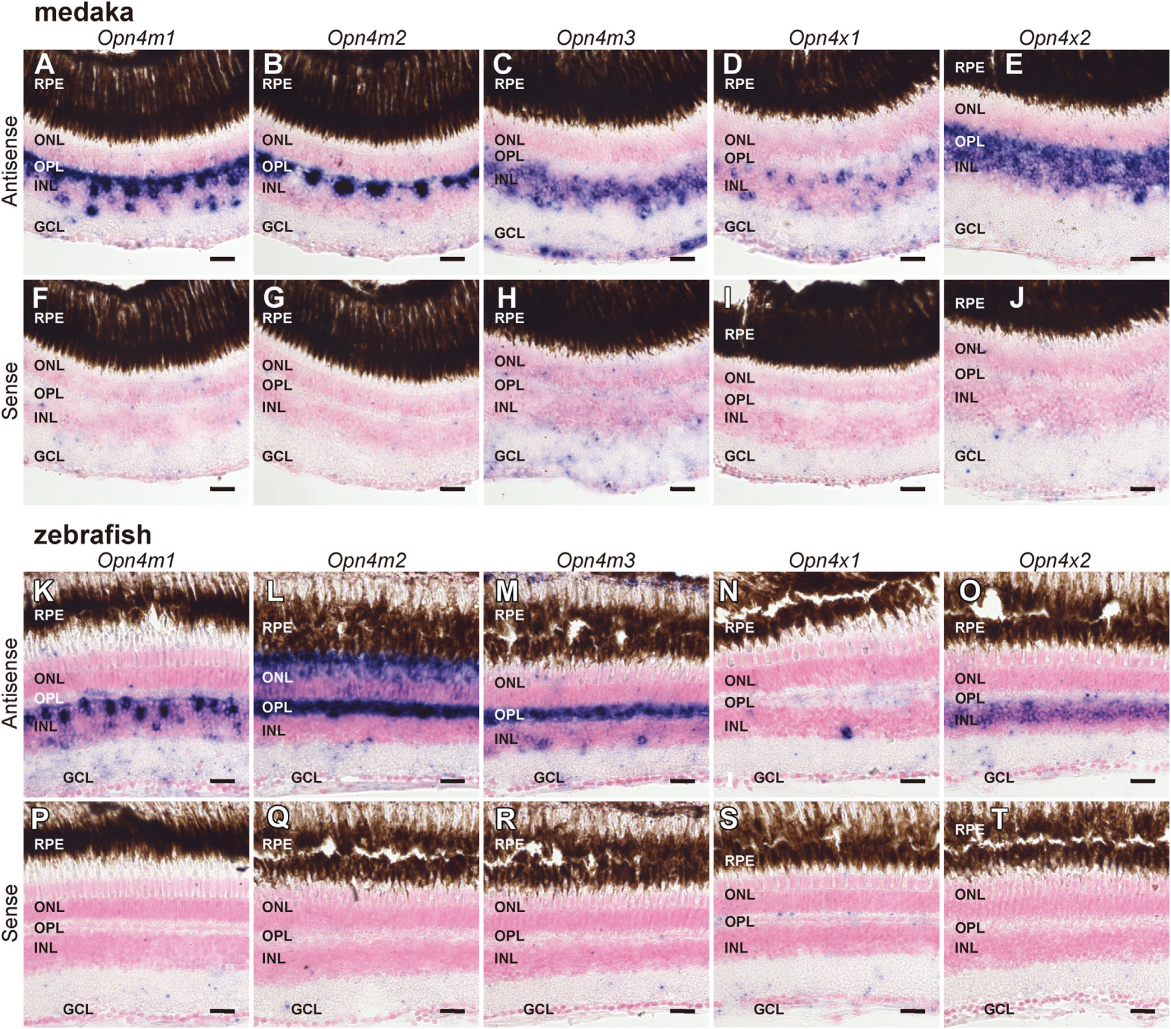
**Distribution of *Opn4* mRNA in the retina of medaka and zebrafish.***A*–*J*, distribution of the transcripts of medaka *Opn4m1* (*A* and *F*), *Opn4m2* (*B* and *G*), *Opn4m3* (*C* and *H*), *Opn4x1* (*D* and *I*) and *Opn4x2* (*E* and *J*) in the retina. *K*–*T*, distribution of the transcripts of zebrafish *Opn4m1* (*K* and *P*), *Opn4m2* (*L* and *Q*), *Opn4m3* (*M* and *R*), *Opn4x1* (*N* and *S*) and *Opn4x2* (*O* and *T*) in the retina. These sections were hybridized with antisense probes (*A*–*E*, *K*–*L*) or corresponding sense probes (*F*–*J*, *P*–*T*). Scale bar: 20 μm.

**Figure 6:**
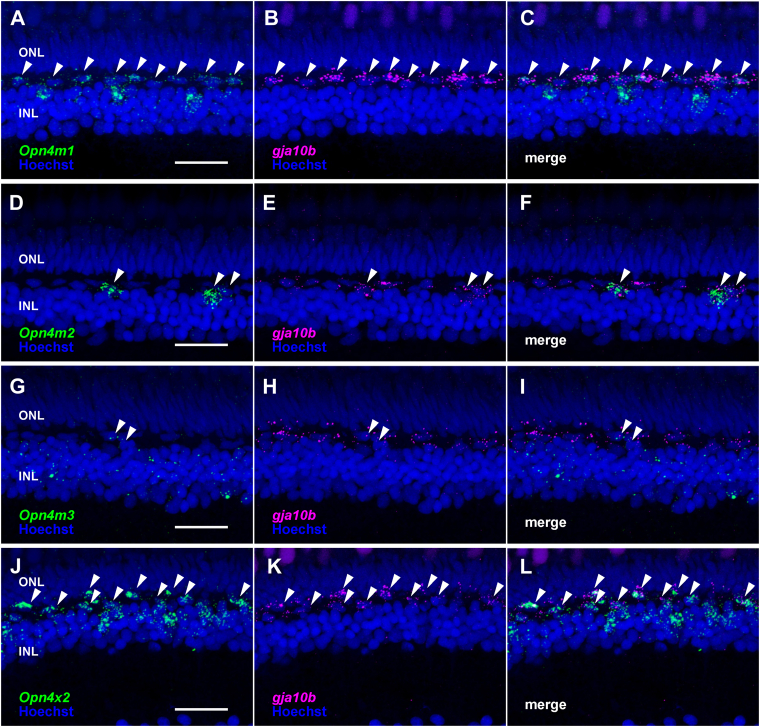
**Distribution of *Opn4* mRNA in the horizontal cells of medaka.***A*–*L*, distribution of the transcripts of medaka *Opn4m1* (*A*–*C*), *Opn4m2* (*D*–*F*), *Opn4m3* (*G*–*I*), and *Opn4x2* (*J*–*L*) and horizontal cell marker (*gja10b*) in the retina. Nuclei were stained with Hoechst (blue). White arrowheads indicate the colocalization of *Opn4* genes and *gja10b* in some cells in the OPL. Scale bar: 20 μm.

Next, we analyzed the distribution patterns of *Opn4* mRNAs in the eyes of medaka and zebrafish in the Teleostei. Among the distribution patterns of medaka *Opn4m* mRNAs in the retina (Fig. 5), the transcript of the intron-containing *Opn4m* gene (*Opn4m1*) was abundant in the cells (presumed horizontal cells) abutting OPL and a small number of the cells on the inner side of the INL (Fig. 5, *A* and *F*). The transcript of the other intron-containing *Opn4m* gene (*Opn4m3*) was distributed in the INL and GCL (Fig. 5, *C* and *H*). On the other hand, the hybridization signals of mRNA of the intron-less *Opn4m* gene (*Opn4m2*) were observed only in a smaller number of the cells (presumed horizontal cells) abutting OPL (Fig. 5, *B* and *G*). These results showed that the distribution patterns of the medaka *Opn4m* mRNAs in the retina were similar to those of the spotted gar *Opn4m* mRNAs. Our double fluorescence *in situ* hybridization analysis confirmed that the expression signals of the horizontal cell marker gene (*gja10b* encoding connexin 52.6) are merged with several *Opn4m*-positive cells abutting the OPL in the medaka retina (Fig. 6, *A*–*I*) (29). In addition, to compare the distribution patterns of *Opn4m* mRNAs between medaka and zebrafish in teleosts, we conducted *in situ* hybridization analysis in the zebrafish retina. Zebrafish *Opn4m2* mRNA showed abundant distribution in the presumed horizontal cells and the photoreceptor cells (Fig. 5, *L* and *Q*), whereas *Opn4m1* mRNA was expressed in a subset of the cells in the INL (Fig. 5, *K* and *P*) and *Opn4m3* mRNA was expressed in the OPL and a small number of cells on the inner side of the INL (Fig. 5, *M* and *R*). These results for zebrafish *Opn4m* genes are consistent with a previous report (19, 20, 30). Thus, the expression patterns of *Opn4m* mRNAs are diversified among teleost species. In addition, the comparison of the localization of *Opn4m2* mRNA in the retina among spotted gar, medaka, and zebrafish showed that the expression patterns of the intron-less *Opn4m* gene changed to be more abundant in zebrafish. We also analyzed the distribution patterns of *Opn4x* mRNAs in the retinas of medaka (Fig. 5, *D*, *E*, *I*, and *J*) and zebrafish (Fig. 5, *N*, *O*, *S*, and *T*). In both teleosts, *Opn4x1* and *Opn4x2* mRNAs were detected in the INL, and the signals of *Opn4x2* mRNA were more abundant than those of *Opn4x1* mRNA. In addition, we confirmed that the horizontal cell marker gene (*gja10b*) is expressed in several *Opn4x2*-positive cells abutting the OPL in the medaka retina (Fig. 6, *J*–*L*). The comparison of the localization of *Opn4x* mRNAs in the retina among spotted gar, medaka, and zebrafish showed that the exclusive expression of the *Opn4x* genes in the INL was conserved among the actinopterygian species.

### Analysis of the expression patterns of *Opn4* mRNA in the brain

**Figure 7:**
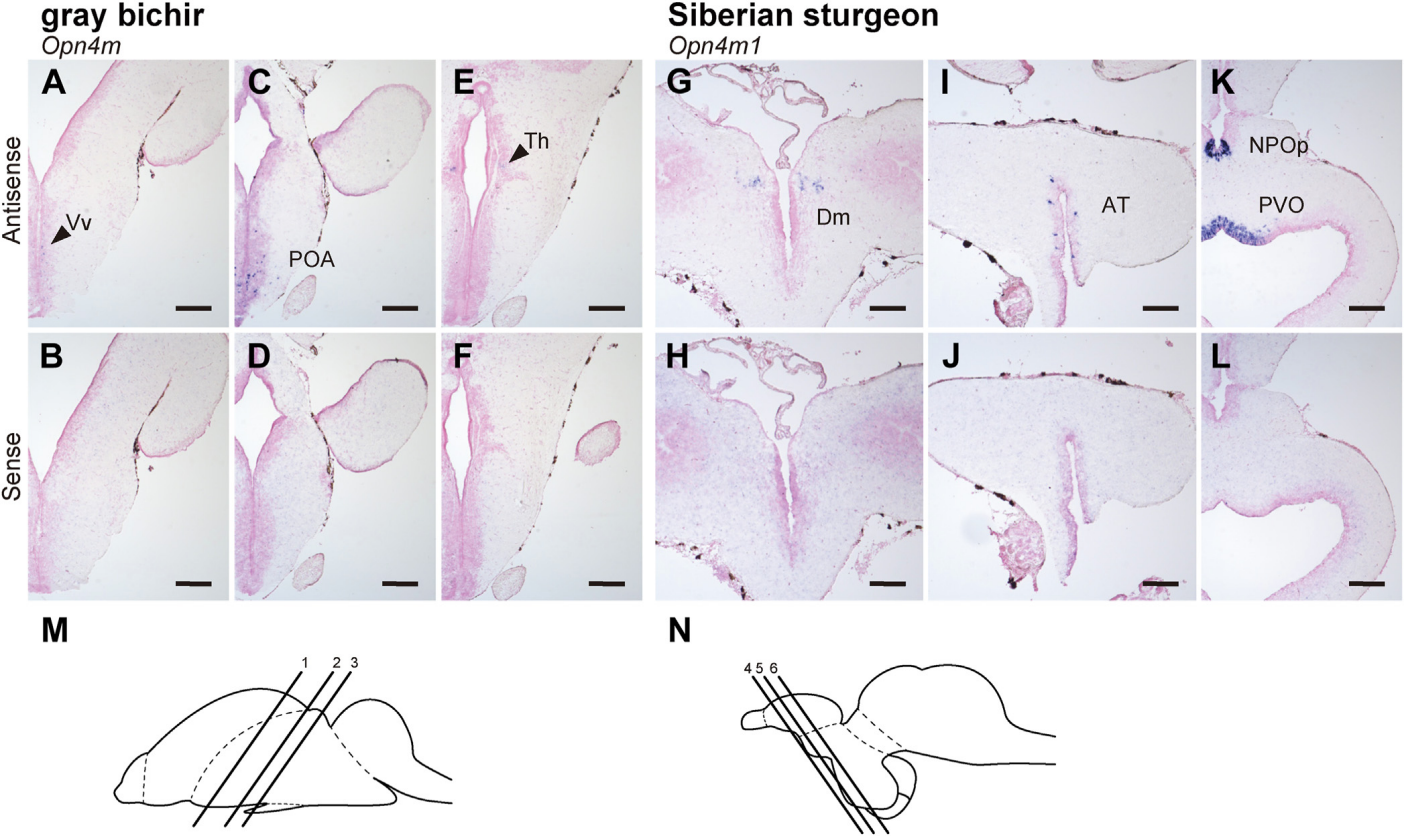
**Distribution of *Opn4* mRNA in the brain of gray bichir and Siberian sturgeon.** (*A–L*) Distribution of the transcripts of gray bichir *Opn4m* (*A*–*F*) and Siberian sturgeon *Opn4m1* (*G*–*L*) in the brain. These sections were hybridized with antisense probes (*A*, *C*, *E*, *G*, *I*, and *K*) or corresponding sense probes (*B*, *D*, *F*, *H*, *J*, and *L*). Scale bar: 200 μm. *M, N*, Schematic drawings of the gray bichir brain (*M*) and Siberian sturgeon brain (*N*) in lateral view. Numbered lines indicate the positions of cross sections shown in *A* and *B* (1), *C* and *D* (2), *E* and *F* (3), *G* and *H* (4), *I* and *J* (5) or *K* and *L* (6).

**Figure 8:**
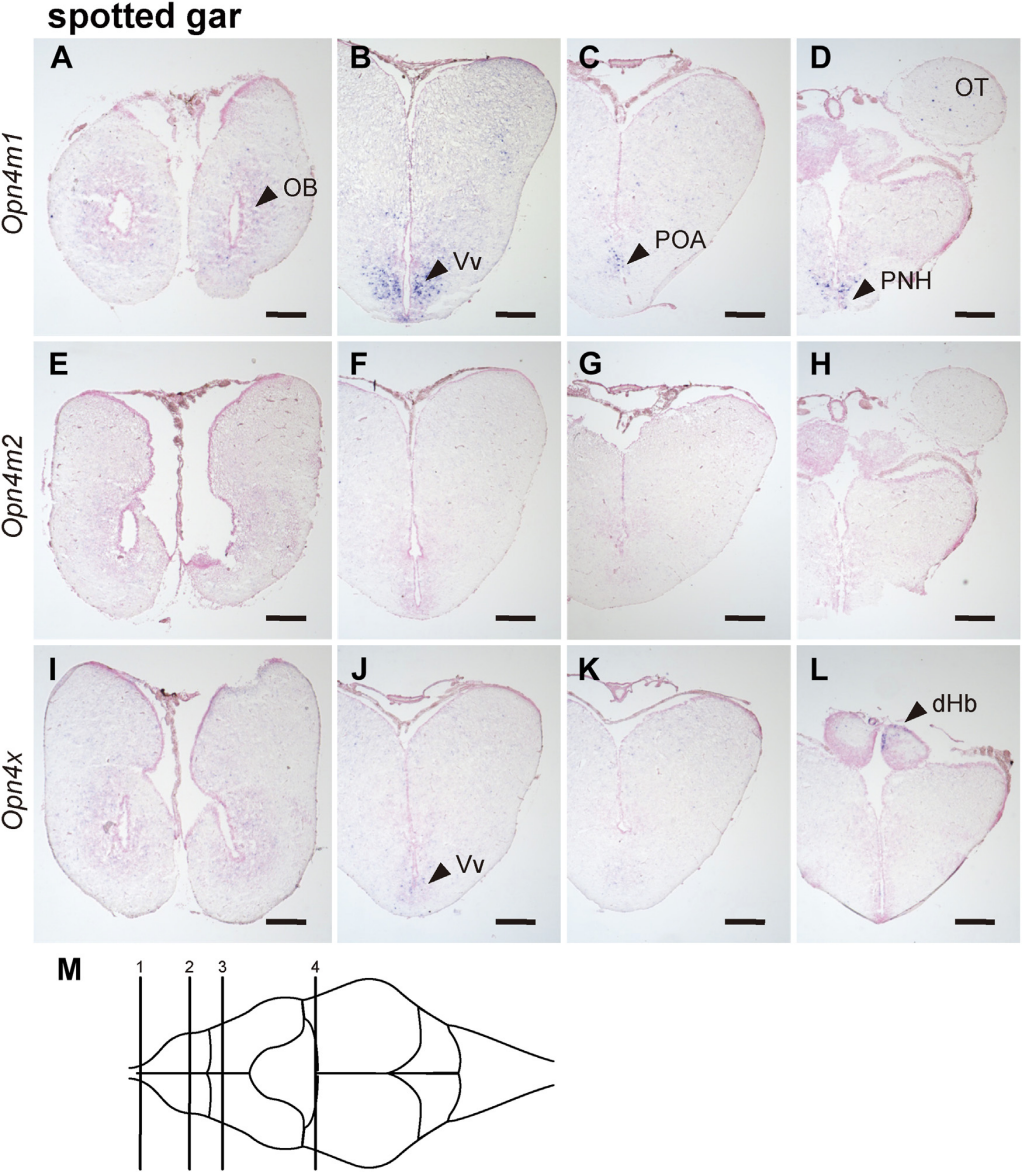
**Distribution of *Opn4* mRNA in the brain of spotted gar.** (*A–L*) Distribution of the transcripts of spotted gar *Opn4m1* (*A*–*D*), *Opn4m2* (*E*–*H*), and *Opn4x* (*I*–*L*) in the brain. These coronal sections were hybridized with antisense probes. The sections which were hybridized with the corresponding sense probes are shown in Fig. S3. Scale bar: 200 μm. (*M*) Schematic drawing of the spotted gar brain in dorsal view. Numbered lines indicate the positions of cross sections shown in (*A*, *E*, and *I*) (1), (*B*, *F*, and *J*) (2), (*C*, *G*, and *K*) (3) or (*D*, *H*, and *L*) (4).

Finally, we analyzed the mRNA distribution of *Opn4* genes within the brains of non-teleost fishes (Fig. 7). We observed the expression signals of these *Opn4* genes in several areas of the telencephalon, diencephalon, and mesencephalon. In the gray bichir brain, *Opn4m* mRNA was expressed in the ventral part of the ventral telencephalic area (Vv) (Fig. 7, *A* and *B*), in the preoptic area (POA), and in a small number of neurons in the thalamus (Th) of the diencephalon (Fig. 7, *C*–*F*). In the Siberian sturgeon brain, *Opn4m1* mRNA was expressed in some parts of the medial part of the dorsal telencephalic area (Dm) and anterior tuberal region (AT) (Fig. 7, *G*–*J*). Moreover, we detected strong hybridization signals of Siberian sturgeon *Opn4m1* mRNA in cerebrospinal fluid-contacting neurons in the parvocellular preoptic nucleus (NPOp) and paraventricular organ (PVO) of the hypothalamus (Fig. 7, *K* and *L*). In the spotted gar telencephalon, diencephalon, and mesencephalon, three *Opn4* genes showed their characteristic expression patterns (Figs. 8 and S3). *Opn4m1* mRNA was distributed in the inner cell layer of the olfactory bulb (OB), POA, the optic tectum (OT), and the periventricular nucleus of the hypothalamus (PNH) (Fig. 8, *A*–*D*). The hybridization signal of spotted gar *Opn4x* mRNA was detected in the dorsal habenula (dHb) (Fig. 8, *I*–*L*). The transcripts of the intron-containing *Opn4* genes, *Opn4m1* and *Opn4x*, were also expressed in the ventral part of the ventral telencephalic area (Vv) in the telencephalon (Fig. 8, *B* and *J*). By contrast, we could not detect the hybridization signals of the transcript of the intron-less *Opn4* gene (*Opn4m2*) in the anterior region of the spotted gar brain (Fig. 8, *E*–*H*).

**Figure 9:**
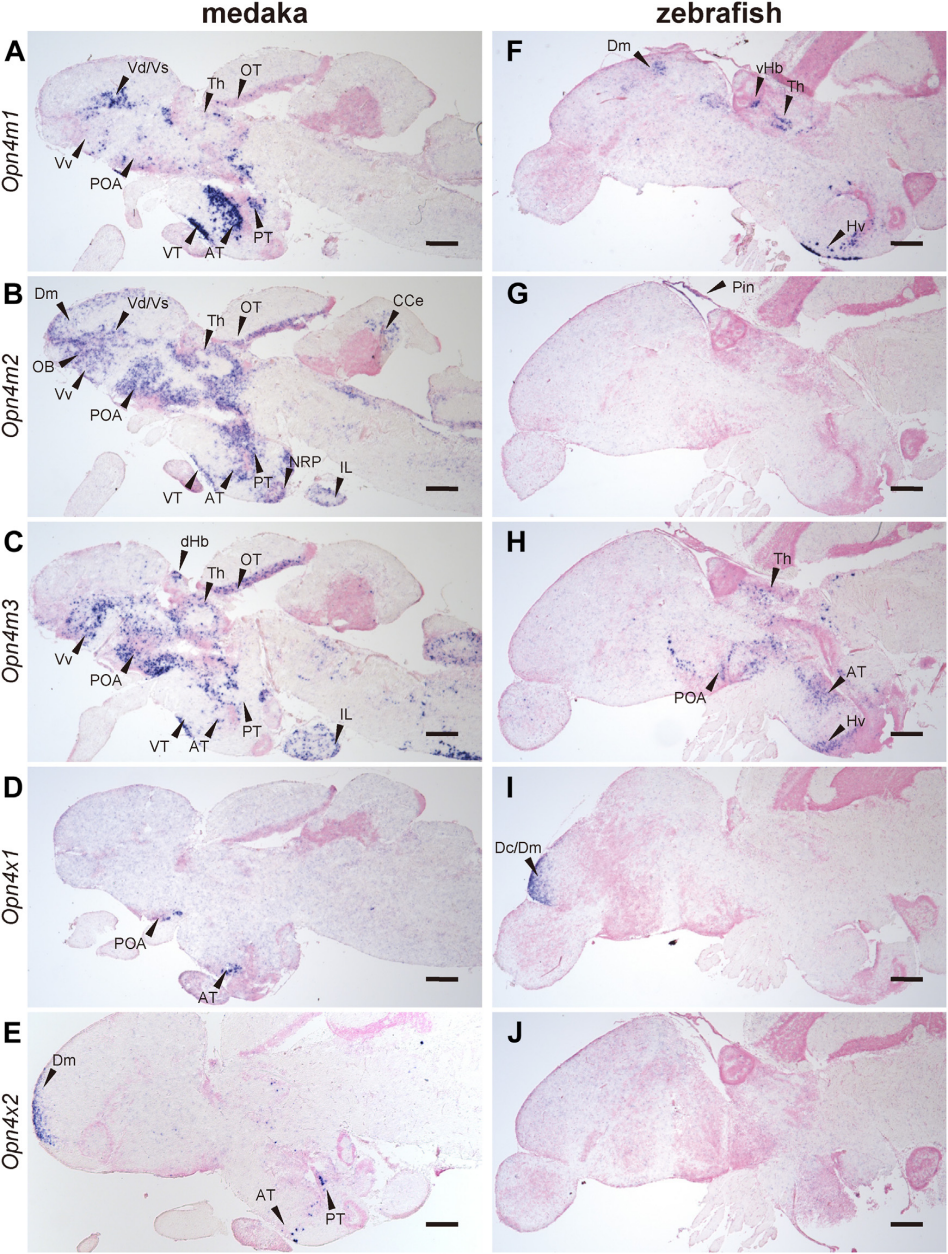
**Distribution of *Opn4* mRNA in the brain of medaka and zebrafish.***A–E*, distribution of the transcripts of medaka *Opn4m1* (*A*), *Opn4m2* (*B*), *Opn4m3* (*C*), *Opn4x1* (*D*), and *Opn4x2* (*E*) in the brain. *F–J*, distribution of the transcripts of zebrafish *Opn4m1* (*F*), *Opn4m2* (*G*), *Opn4m3* (*H*), *Opn4x1* (*I*), and *Opn4x2* (*J*) in the brain. These sagittal sections were hybridized with antisense probes. The sections which were hybridized with the corresponding sense probes are shown in Fig. S4. Scale bar: 200 μm.

To compare the expression patterns of *Opn4* mRNAs between teleost and non-teleost species, we also analyzed the distribution of *Opn4* mRNAs in the brains of medaka and zebrafish (Figs. 9 and S4). We observed the distribution of *Opn4m* mRNAs in a large area within the medaka brain (Fig. 9, *A*–*C*). Notably, the transcript of the medaka intron-less *Opn4m* gene (*Opn4m2*) was expressed in OB, Dm, dorsal part of the ventral telencephalic area (Vd), supracommisural part of the ventral telencephalic area (Vs), Vv, POA, Th, OT, ventral tuberal region (VT), anterior tuberal region (AT), posterior tuberal region (PT), nucleus recessus posterioris (NRP), inferior lobe (IL), and cerebellar corpus (CCe) (Fig. 9*B*). Medaka *Opn4m1* mRNA was detected in Vd, Vs, Vv, POA, VT, AT, and PT. The expression signals of the other intron-containing *Opn4m* gene (*Opn4m3*) in medaka were observed in Vv, POA, dHb, Th, OT, VT, AT, PT, and IL. In the zebrafish brain, *Opn4m2* mRNA was detected only in the pineal gland (Pin) (Fig. 9*G*), whereas the intron-containing *Opn4* genes (*Opn4m1* and *Opn4m3*) were expressed in several parts of the brain including Dm, POA, the ventral habenula (vHb), Th, AT and the ventral zone of the periventricular hypothalamus (Hv) (Fig. 9, *F* and *H*). A previous study reported the significantly low expression level of the *Opn4* gene in the zebrafish brain (6), which is consistent with our results. The comparison of the localization of *Opn4m2* mRNA in the brain among spotted gar, medaka, and zebrafish showed that the expression patterns of the intron-less *Opn4m* gene changed to be more abundant in some teleost lineages. Moreover, the *Opn4x* genes showed different expression patterns in the brain between medaka and zebrafish. In the rostral surface of Dm, the expression signals of medaka *Opn4x2* mRNA, but not *Opn4x1*, were observed (Fig. 9, *D* and *I*). By contrast, in the corresponding region of the zebrafish brain, the hybridization signals of *Opn4x1* mRNA, but not *Opn4x2*, were detected (Fig. 9, *E* and *J*). Medaka *Opn4x1* and *Opn4x2* mRNAs were also detected in POA, AT, and PT, although zebrafish *Opn4x1* and *Opn4x2* mRNAs were not observed in regions other than Dm. Collectively, these results indicated that the expression patterns of *Opn4* genes in the brain were diversified among the actinopterygian species.

## Discussion

**Figure 10:**
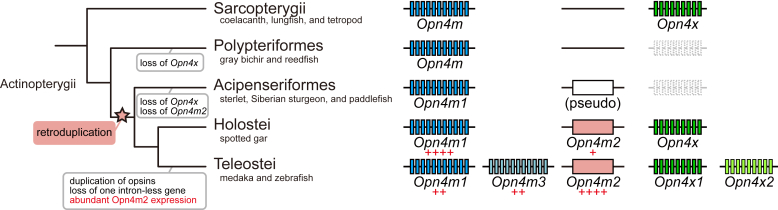
**Evolutionary model of teleost intron-containing and intron-less *Opn4* genes in the Actinopterygii.** It can be speculated that the ancestor of the Actinopterygii had two *Opn4* genes, *Opn4m* and *Opn4x*, both of which contained introns. After branching of the Polypteriformes, the intron-less *Opn4m* gene (*Opn4m2*) emerged by retroduplication of the parental intron-containing *Opn4m* gene (*Opn4m*). However, this newly acquired intron-less *Opn4m* gene was pseudogenized in the Acipenserformes. In addition, the *Opn4x* gene was lost independently in the Polypteriformes and the Acipenserformes. The common ancestor of the Holostei and the Teleostei had three *Opn4* genes (*Opn4m1*, *Opn4m2* and *Opn4x*) and subsequently, in the Teleostei lineage, the intron-containing *Opn4* genes (*Opn4m1* and *Opn4x*) duplicated by a whole genome duplication. In addition, an increase of the cells which express the intron-less *Opn4m* gene (*Opn4m2*) in the retina or brain occurred in several teleost species.

In this study, to assess the diversification process of the intron-containing and intron-less *Opn4* genes in the Actinopterygii, we analyzed the synteny of the *Opn4* genes in both teleost and non-teleost fishes. Based on our analysis, we propose an evolutionary model of *Opn4* genes in the Actinopterygii (Fig. 10). The ancestor of the Actinopterygii had two *Opn4* genes, *Opn4m* and *Opn4x*, both of which contained introns. After branching of the Polypteriformes, the intron-less *Opn4m* gene (*Opn4m2*) emerged by retroduplication of the parental intron-containing *Opn4m* gene (*Opn4m*). The common ancestor of the Holostei and the Teleostei had three *Opn4* genes (*Opn4m1*, *Opn4m2*, and *Opn4x*), and subsequently, in the Teleostei lineage, the intron-containing *Opn4* genes (*Opn4m1* and *Opn4x*) duplicated by whole genome duplication. In addition, our analysis of the molecular properties of Opn4 proteins showed that Opn4 proteins maintained the sensitivity to blue light and the coupling to Gq-type G protein during the diversification process in the Actinopterygii. Also, the comparison of the distribution patterns of *Opn4* mRNA showed that the newly acquired intron-less *Opn4m* gene (*Opn4m2*) was expressed in a quite-restricted area of the retina in spotted gar, whereas the orthologous gene was expressed in a large area of the anterior region of the brain in medaka and expressed abundantly in the horizontal cells and the photoreceptor cells of the retina in zebrafish. This showed that the intron-less *Opn4m* gene expanded its expression areas in the retina or brain in the Teleostei lineage. As mentioned in Figure 1, it can be speculated that the intron-less *rhodopsin* gene (*Rh1-2*) emerged after the branching of the Polypteriformes and became predominantly used in the retina without substantial changes of the molecular property of the rhodopsin protein in the Teleostei lineage. Therefore, our analysis of the *rhodopsin* and *Opn4* genes in the Actinopterygii suggested that the teleost intron-less *rhodopsin* and *Opn4* genes coincidently experienced similar evolutionary events after their emergence by retroduplication.

The comparison of the synteny of the *rhodopsin* and *Opn4* genes also unveiled a loss of the opsin genes in several lineages. In the Acipenseriformes, paddlefish has one intron-less *rhodopsin* protein-coding gene together with one intron-less *rhodopsin* pseudogene which was speculated to have been pseudogenized after a whole genome duplication in this lineage. In addition, sterlet and paddlefish have one or two intron-containing *Opn4* protein-coding gene(s) together with two intron-less *Opn4* pseudogenes. These pseudogenes share the same synteny and nonsense mutation position (Fig. 2*B*). This suggests that the intron-less *Opn4* genes of sterlet and paddlefish were pseudogenized in their common ancestor before the divergence of the Acipenseridae and Polyodontidae in the Acipenseriformes (153 Mya) (21). These gene losses are thought to be driven by the functional redundancy of opsins. The intron-containing *rhodopsin* gene (*Rh1*) in gray bichir and the intron-less *rhodopsin* gene (*Rh1-2*) in Siberian sturgeon exhibit similar expression patterns, exclusive and abundant expression in the photoreceptor cells of the retina, and encode proteins that have similar spectral and biochemical properties (13). The intron-containing *Opn4m* gene also has generally conserved expression patterns in gray bichir and Siberian sturgeon, although the Siberian sturgeon *Opn4m* gene (*Opn4m1*) shows several specific expressions such as in the RPE cells of the retina and the cerebrospinal fluid-contacting neurons of the brain. Also, Opn4 proteins encoded by the intron-containing *Opn4m* genes in gray bichir and Siberian sturgeon share spectral and biochemical properties. These functional redundancies of opsins would have led to a random loss of the intron-containing or the intron-less opsin gene in the Acipenseriformes.

In our results of *in situ* hybridization analysis, we observed that teleost *Opn4* genes show more widespread expression patterns in the brain than non-teleost *Opn4* genes. However, we found that *Opn4* genes in the Actinopterygii show several common distribution patterns in the retina and the brain, such as expression in the telencephalon and diencephalon. For example, all actinopterygian species we analyzed speculated to express the transcripts of the *Opn4* genes in the horizontal cells of the retina. These results suggest that the Opn4-mediated photoreception in the horizontal cells is well conserved among the Actinopterygii (30). In addition, in all actinopterygian species analyzed in this study, we detected the expression signals of the *Opn4* genes in the POA, where several other opsin genes such as the *Opn3/tmt-opsins* and *Opn5* genes in the Actinopterygii have been reported to be expressed (31, 32). Also, several studies showed that Opn4 proteins in the zebrafish brain are involved in the regulation of locomotor activity in the larva (33, 34). Further comparative investigations will reveal the functional similarities and differences of the *Opn4* genes among actinopterygian species and lead to the understanding of the physiological relevance of the presence of multiple *Opn4* genes in the Actinopterygii.

In addition to the *rhodopsin* and *Opn4* genes in the Actinopterygii, it has been reported that Cyprinodontiformes fishes have a retrogene (*S180r*) of long wavelength sensitive (*LWS*) cone visual pigments (11, 35). For example, guppy (*Poecilia reticulate*) has four *LWS* opsin genes, among which one is a retrogene (two exons) duplicated from a parental *LWS* opsin gene (six exons). The sequence comparison of the LWS opsins encoded by the retrogenes in the Cyprinodontiformes shows that guppy LWS-4 protein in the Poeciliidae has a histidine residue at position 181 (based on the bovine rhodopsin numbering system), whereas sheepshead minnow (*Cyprinodon variegatus*) LWS-4 protein in the Cyprinodontidae has a tyrosine residue at this position (Fig. S5) (36). Most LWS opsins in vertebrates have His181, which participates in the binding of Cl^-^ to induce the red shift of λmax (37). By contrast, the opsins in the Glires, such as mouse (*Mus musculus*) in the Rodentia and rabbit (*Oryctolagus cuniculus*) in the Lagomorpha, have Tyr181, which is responsible for their blue-shifted λmax (38, 39, 40). This H181Y mutation has rarely been found in the opsins of species other than the Glires. Thus, it can be speculated that this unique mutation in the retrogene results in a blue shift of λmax of the LWS-4 protein and contributes to the expansion of the spectral sensitivities covered by the LWS proteins in the Cyprinodontidae.

In conclusion, we propose an evolutionary model of the teleost intron-less *Opn4* gene, that is, the intron-less *Opn4* gene emerged after branching of the Polypteriformes and became abundantly used in the Teleostei lineage. This stepwise evolutionary model is common with that of the teleost intron-less *rhodopsin* gene, which would have contributed to the different repertoires of the opsin genes and the diversity of the photoreceptive functions in the Actinopterygii. It should be noted that the intron-containing and intron-less genes of *rhodopsin* and *Opn4* show several similar expression patterns in the retinas of actinopterygian species, such as *rhodopsin* genes in the rod cells and *Opn4m* genes in the cells abutting the OPL in the spotted gar retina. This suggests the possibility that these retrogenes were formed by the reverse transcription of mRNAs including the upstream regions of their parental genes (12). In fact, a previous study reported that the upstream sequences of intron-containing and intron-less *rhodopsin* genes of zebrafish share several common cis-elements responsible for the transcriptional regulation of the retinal genes (41). Further comparative analysis of the *cis*-regulation mechanisms between the intron-containing and intron-less opsin genes based on the accumulated genomic information will reveal the detailed evolutionary process of *rhodopsin* and *Opn4* genes in the Actinopterygii.

## Experimental procedures

### Animals and ethics statement

Gray bichir (*P. senegalus*: ∼15 cm), Siberian sturgeon (*A. baerii*: ∼20 cm), and spotted gar (*L. oculatus*: ∼7 cm) were purchased from a local pet shop. Zebrafish (*Danio rerio*) was purchased from Kamihata Fish Industries Ltd. Medaka (*Oryzias latipes*) was maintained and bred at Kyoto University. The use of animals in these experiments was in accordance with guidelines established by the Ministry of Education, Culture, Sports, Science and Technology of Japan. The protocols in this paper were approved by the Animal Care and Use Committee of Kyoto University (permit number: H2919, H3015, 201913, 202012 and 202109).

### Isolation of cDNA encoding opsin

To isolate the clones of gray bichir, spotted gar, zebrafish, and medaka *Opn4*, we searched them in the genomic databases and identified their full-length ORF sequences. The clones of gray bichir *Opn4m* (accession no. XM_039768409.1), spotted gar *Opn4m1* (XM_006630896.2), *Opn4m2* (LC743797) and *Opn4x* (XM_015340274.1), zebrafish *Opn4m1* (GQ925715.1), *Opn4m2* (GQ925716.1), *Opn4m3* (GQ925717.1), *Opn4x1* (GQ925718.1) and *Opn4x2* (GQ925719.1) and medaka *Opn4m1* (XM_023963602.1), *Opn4m2* (XM_011486336.3), *Opn4m3* (XM_020712260.2), *Opn4x1* (XM_011481794.2), and *Opn4x2* (XM_023958407.1) were isolated by PCR from the first strand cDNA of the eyes or brain as shown in our previous studies (13, 42). To isolate the clone of Siberian sturgeon *Opn4m1*, we first searched it in the transcriptome data deposited at the NCBI Sequence Read Archive (BioProject accession ID: SRX2426785). We successfully identified sequence reads including the start codon and stop codon of *Opn4m1* ORF. Based on these sequence reads, we performed RT-PCR on eye RNA and isolated the full-length ORF sequence of Siberian sturgeon *Opn4m1* (LC743796).

### Preparation of Opn4 recombinant proteins

To improve the expression level of Opn4 recombinant proteins, we conducted C-terminus truncation according to our previous study on mouse Opn4 (23). We truncated 199 amino acid residues from the C-terminus of gray bichir Opn4m, 186 residues from Siberian sturgeon Opn4m1, 192 residues from spotted gar Opn4m1, 110 residues from spotted gar Opn4m2, 189 residues from spotted gar Opn4x, 205 residues from zebrafish Opn4m1, 125 residues from zebrafish Opn4m2, 184 residues from zebrafish Opn4m3, 198 residues from zebrafish Opn4x1, 132 residues from zebrafish Opn4x2, 167 residues from medaka Opn4m1, 96 residues from medaka Opn4m2, 157 residues from medaka Opn4m3, 79 residues from medaka Opn4x1, and 216 residues from medaka Opn4x2, which correspond to the deletion region of mouse Opn4 shown in our previous paper (23). The C-terminal truncated *Opn4* cDNAs were tagged with the epitope sequence of the anti-bovine rhodopsin monoclonal antibody Rho1D4 (ETSQVAPA) at the C-terminus and were introduced into the mammalian expression vector pCAGGS (43). We also prepared the full-length cDNAs with the Rho1D4 epitope sequence and the C-terminal truncated cDNAs without the Rho1D4 epitope sequence for spotted gar Opn4m1, Opn4m2, and Opn4x. The plasmid DNA was transfected into HEK293S cells, which were maintained at 37 °C with 5% CO_2_, using the calcium phosphate method as previously described (23). 11-*cis* retinal was added to the medium (final concentration: 5 μM) 24 h after transfection. The cells were kept in the dark before they were collected at 48 h after transfection. The reconstituted pigments were extracted from cell membranes with 1% DDM in Buffer A (50 mM HEPES, 140 mM NaCl, pH 6.5). To remove other extract components from the DDM-solubilized sample, the elution was incubated with Rho1D4-conjugated agarose overnight and washed with 0.02% DDM and 20% glycerol (v/v) in Buffer A. The Opn4 pigments were eluted with 0.02% DDM and 20% glycerol in Buffer A containing the synthetic peptide that corresponds to the C-terminus of bovine rhodopsin. We carried out all procedures on ice under dim red light.

### Spectroscopic measurements

UV/Vis absorption spectra were recorded using a spectrophotometer (UV2400, UV2450 or UV2600, Shimadzu) and an optical cell (width, 2 mm; light path, 1 cm). The sample temperature was maintained using a temperature controller (RTE-210, NESLAB) at 0 ± 0.1 °C. The sample was irradiated with light which was generated by a 1-kW tungsten halogen lamp (Master HILUX-HR, Rikagaku Seiki) and passed through an optical filter (Y-52, AGC Techno Glass).

### Ca^2+^ level measurement in cultured cells

Ca^2+^ levels in cultured cells were measured using an aequorin-based bioluminescence assay (44, 45). To identify the effect of the C-terminus modifications on G protein signaling, we additionally prepared full-length cDNAs with 1D4 epitope (FL-1D4) and C-terminus truncated cDNAs without 1D4 epitope (CT-no1D4) encoding spotted gar Opn4m1, Opn4m2, and Opn4x proteins and were introduced into the mammalian expression vector pCAGGS. HEK293S cells were seeded in 96-well plates at a density of 60,000 cells/well in medium (D-MEM/F12 containing 10% FBS). After incubation for 24 h, the plasmid DNA was transfected into the cells (100 ng/well) using the polyethyleneimine transfection method. After incubation for 6 h, 11-*cis* retinal was added to the medium (final concentration: 5 μM). After overnight incubation, the medium was replaced with the equilibration medium (CO_2_-independent medium containing coelenterazine h and 10% FBS). Following equilibration for 2 h at room temperature, luminescence from the cells was measured using a microplate reader (SpectraMax L, Molecular Devices). The cells were irradiated for 5 s with blue LED light (450 nm) to trigger the change of the luminescence.

### *In situ* hybridization

Tissue sample preparation and *in situ* hybridization were performed according to a previous paper (32) with slight modifications. The fish were euthanized by immersion in 0.04% MS-222 and immediate decapitation. After the eyes and brains were dissected from those fish, they were fixed overnight in PBS containing 4% PFA at 4 °C. The tissues were then cryoprotected by soaking overnight in PBS containing 20% sucrose, embedded in OCT compound, and frozen at −80 °C. Frozen tissues were sliced into 16-μm sections and affixed to glass slides. They were stored at −20 °C until use.

Digoxigenin-labeled riboprobes of *Opn4* genes were synthesized from full-length cDNAs flanked by T7 and T3 promoter sequences which were inserted into pBluescript II KS (+) or pTA2 (TOYOBO). All of the following procedures were performed at room temperature (RT) unless otherwise noted. Tissue sections were sequentially immersed in 4% PFA in PBS for 15 min, 100% methanol for 30 min, PBS for 5 min, Proteinase K (0.5 μg/ml)/Tris-EDTA buffer (50 mM Tris-HCl, 5 mM EDTA, pH 7.6) for 15 min, 4% PFA in PBS for 15 min, dimethyl dicarbonate-treated water for 30 s, acetylation buffer (0.27% (v/v) acetic anhydride, 100 mM triethanolamine, pH 8.0) for over 10 min, PBS for 5 min, and hybridization buffer (750 mM NaCl, 75 mM sodium citrate, 0.4 mg/ml yeast RNA, 0.1 mg/ml heparin sodium, 1 × Denhardt's solution, 0.1% (v/v) Tween, 0.1% (w/v) CHAPS, 5 mM EDTA, 70% (v/v) formamide) at 65 °C for 3 h. Digoxigenin-labeled riboprobes (final concentration: 0.17 μg/ml) diluted in hybridization buffer were then applied to tissue sections and incubated at 65 °C for approximately 40 h.

After hybridization, the tissue sections were washed in 1 × SSC buffer (150 mM NaCl, 15 mM sodium citrate, pH 7.0) containing 50% formamide at 65 °C for 15 min and 1 h, followed by washing in 0.2 × SSC buffer at 65 °C for 1 h and MABT (100 mM maleate, 150 mM NaCl, 0.1% Tween 20, pH 7.5) three times for 30 min each. After washing, tissue sections were soaked in blocking buffer (1% (w/v) bovine serum albumin, 10% (v/v) sheep normal serum, 0.08% (v/v) Triton-X100 in PBS) for 30 min and then incubated with alkaline phosphatase-conjugated anti-digoxigenin Fab fragment (1:2000 dilution; Roche) overnight at 4 °C.

Tissue sections were then rinsed three times for 30 min each with MABT and twice for 5 min each with AP reaction buffer (100 mM Tris-HCl, 50 mM MgCl_2_, 100 mM NaCl, and 0.1% Tween 20, pH 9.5) for 5 min each. Color development was performed with 50 μg/ml nitro blue tetrazolium, 175 μg/ml 5-bromo-4-chloro-3-indolyl phosphate, 1 mM levamisole hydrochloride, and 5% (w/v) polyvinyl alcohol in AP buffer at 28 °C for 2 days. Tissue sections were counterstained with Nuclear Fast Red, dehydrated with 70 and 100% Tissue Dehydration Solution (FUJIFILM Wako Pure Chemical), and cleared by immersion in G-NOX (Genostaff). After clearing, the tissue sections were coverslipped with PARAmount mounting medium (FALMA).

### Fluorescence *in situ* hybridization

The mRNA of medaka *opn4* and *gja10b* were detected using signal amplification by exchanging reaction (SABER) with single-strand DNA probes and branches extended by primer exchanging reaction according to the original paper (46) with slight modifications. Probe sequences were designed using OligoMiner software (47). The synthesized single strand DNA probes and branches were confirmed to be 400 to 700 nucleotides in length and purified using DNA Clean & Concentrator-25 (Zymo Research).

The tissue sections were immersed in PBST (PBS containing 0.1% Tween 20), treated with 0.05 mg/ml pepsin in 0.01 M HCl at 37 °C for 20 min, and washed with PBST three times for 5 min each. The following hybridization and washing steps were repeated once for probes and twice for branch DNAs: The tissue sections were immersed in Whyb (0.3 M NaCl, 30 mM sodium citrate, 40% deionized formamide, pH 7.0) buffer for 10 min. Probes or branches diluted with Hyb1 (Whyb buffer containing 10% dextran sulfate) buffer were applied onto the sections at 1 μg/ml and incubated for 16 h. After hybridization, the tissue sections underwent a series of washes with Whyb for 30 min twice, 2 × SSC buffer containing 0.1% Tween 20 for 5 min twice. These steps were performed at 43 °C for probes and 37 °C for branches. After these iterations, the tissue sections were rinsed twice with PBST at RT, incubated with oligo DNAs labeled by fluorophore diluted in PBST at 0.1 μM at 37 °C for 1 h, rinsed twice with PBST again, and counterstained with 1 μg/ml Hoechst 33342 in PBST. Finally, coverslips were mounted on glass slides with homemade PVA/glycerol mounting medium.

Confocal fluorescence images were collected with a laser scanning confocal microscope system, Zeiss LSM 780 (Carl Zeiss Microscopy GmbH, Oberkochen, Germany) with 405, 488, and 561 nm laser lines at the Central Research Laboratory, Okayama University Medical School. Confocal z-stack images were acquired at 0.555 μm intervals. Confocal fluorescence images were manipulated by ZEN 2012 SP1 black edition (64 bit, version 8.1) and ImageJ (48). DNA sequences of probes, branches, hairpins, and oligos labeled by fluorophore, and combinations of applied probes and branches in the above procedure were summarized in Table S1.

## Data availability

All data are available in the main text or the supplementary materials. The sequences reported in this paper have been deposited in the GenBank database (accession numbers LC743796 and LC743797).

## Supporting information

This article contains supporting information (37, 38, 39, 40).

## Conflict of interest

The authors declare that they have no conflicts of interest with the contents of this article.
