# Supplementary material for: Diversification processes of teleost intron-less opsin genes
Source: J Biol Chem. 2023 Jun 7;299(7):104899. doi: 10.1016/j.jbc.2023.104899 (PMC10339062; doi:10.1016/j.jbc.2023.104899)
Supplement: Supporting information [file mmc3.pdf]

Supporting information for

Diversification processes of teleost intron-less opsin genes

Authors:

Chihiro Fujiyabu [1], Keita Sato [2], Hideyo Ohuchi [2], Takahiro Yamashita [1*]

Affiliations:

[1] Department of Biophysics, Graduate School of Science, Kyoto University, Kyoto 606-8502, Japan; [2] Department of Cytology and Histology, Okayama University Faculty of Medicine, Dentistry and Pharmaceutical Sciences, Okayama 700-8558, Japan.

*Corresponding Author:

Takahiro Yamashita, Department of Biophysics, Graduate School of Science, Kyoto University, Kyoto 606-8502, Japan.

Email: yamashita.takahiro.4z@kyoto-u.ac.jp

Supplementary Figure 1

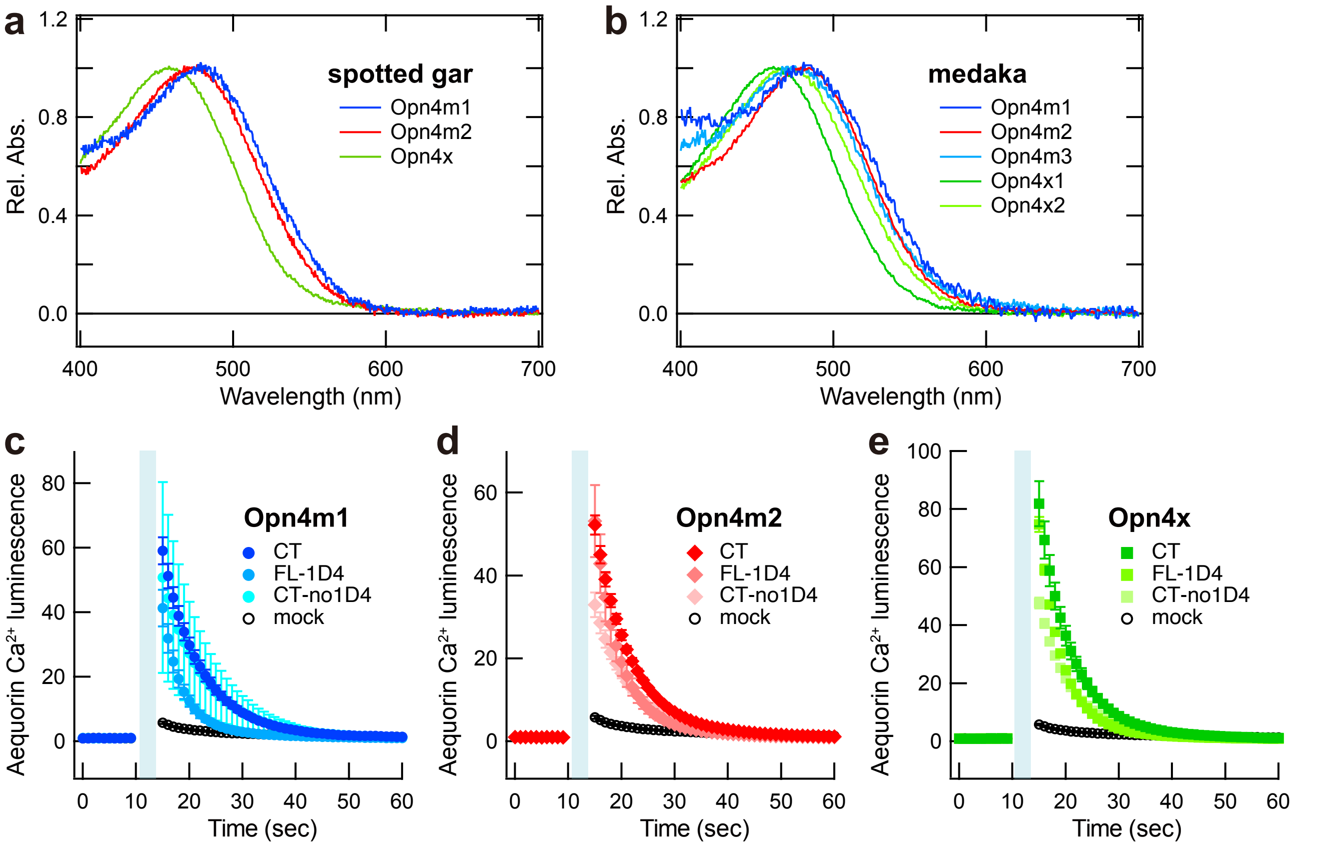

Supplementary Figure 1 Molecular characteristics of Opn4 proteins

a Absorption spectra of spotted gar Opn4m1 protein (blue), Opn4m2 protein (red) and Opn4x protein (green). We conducted affinity column chromatography to remove other extract components from DDM-solubilized extractions of spotted gar Opn4 proteins and measured their absorption spectra. λmax of Opn4m1, Opn4m2 and Opn4x proteins were estimated to be 482 nm, 477 nm, and 461 nm, respectively. **b** Absorption spectra of medaka Opn4m1 protein (blue), Opn4m2 protein (red), Opn4m3 protein (light blue), Opn4x1 protein (green) and Opn4x2 protein (light green). λmax of Opn4m1, Opn4m2, Opn4m3, Opn4x1 and Opn4x2 proteins were estimated to be 487 nm, 482 nm, 480 nm, 463 nm, and 474 nm, respectively. **c-e** Light-induced changes of intracellular Ca^{2+} level by spotted gar Opn4 proteins. The Ca^{2+} level in the HEK293S cells expressing the C-terminal truncated sequence with the Rho1D4 epitope (CT, same data as shown in Fig. 3g), the full-length sequence with the Rho1D4 epitope (FL-1D4) and the C-terminal truncated sequence without the Rho1D4 epitope (CT-no1D4) of spotted gar Opn4m1 (**c**), Opn4m2 (**d**), or Opn4x (**e**) protein was measured using an aequorin-based luminescent assay. Ca^{2+}-dependent luminescence change of aequorin was triggered by light irradiation from blue (450 nm) LED for 5 sec. Data are presented as the means ± deviations of two independent experiments compared with those of mock-transfected cells.

Supplementary Figure 2

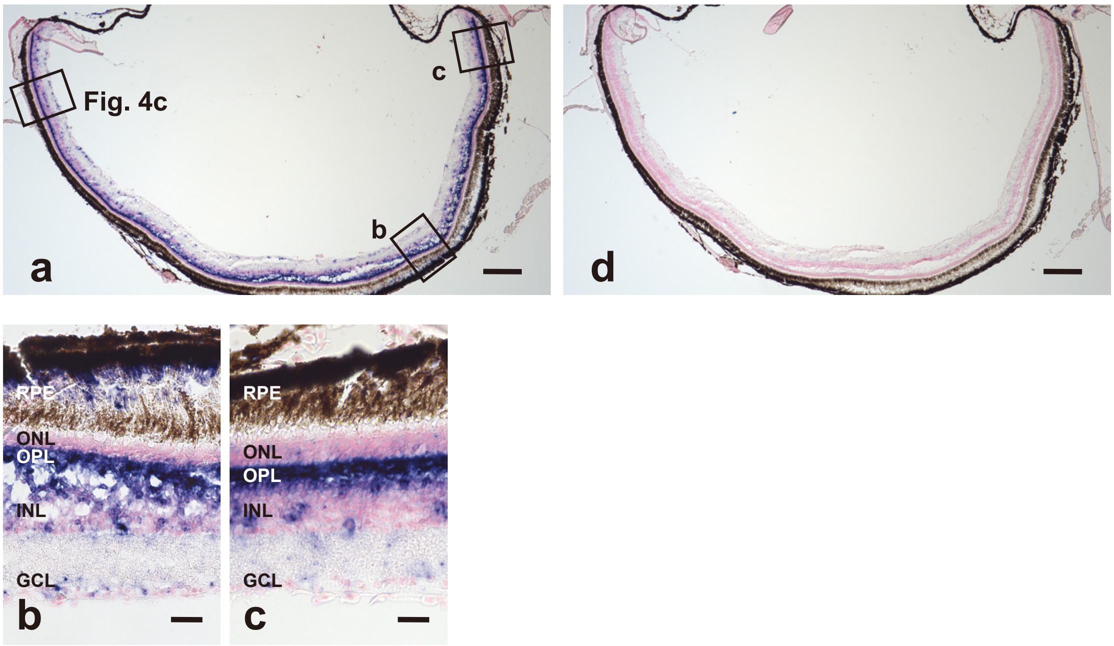

Supplementary Figure 2 Distribution patterns of *Opn4m1* mRNA in the eye of spotted gar The hybridization signals of spotted gar *Opn4m1* mRNA were detected in the RPE cells in the fundus but not in the ventral or dorsal area. The sections were hybridized with antisense probes (**a-c**) or corresponding sense probes (**d**). Enlarged views of the fundus and dorsal regions in (**a**) are shown in (**b**) and (**c**), respectively. Enlarged view of the ventral region is shown in Fig.4c. Scale bar: 200 μm (**a,d**) and 20 μm (**b,c**).

Supplementary Figure 3

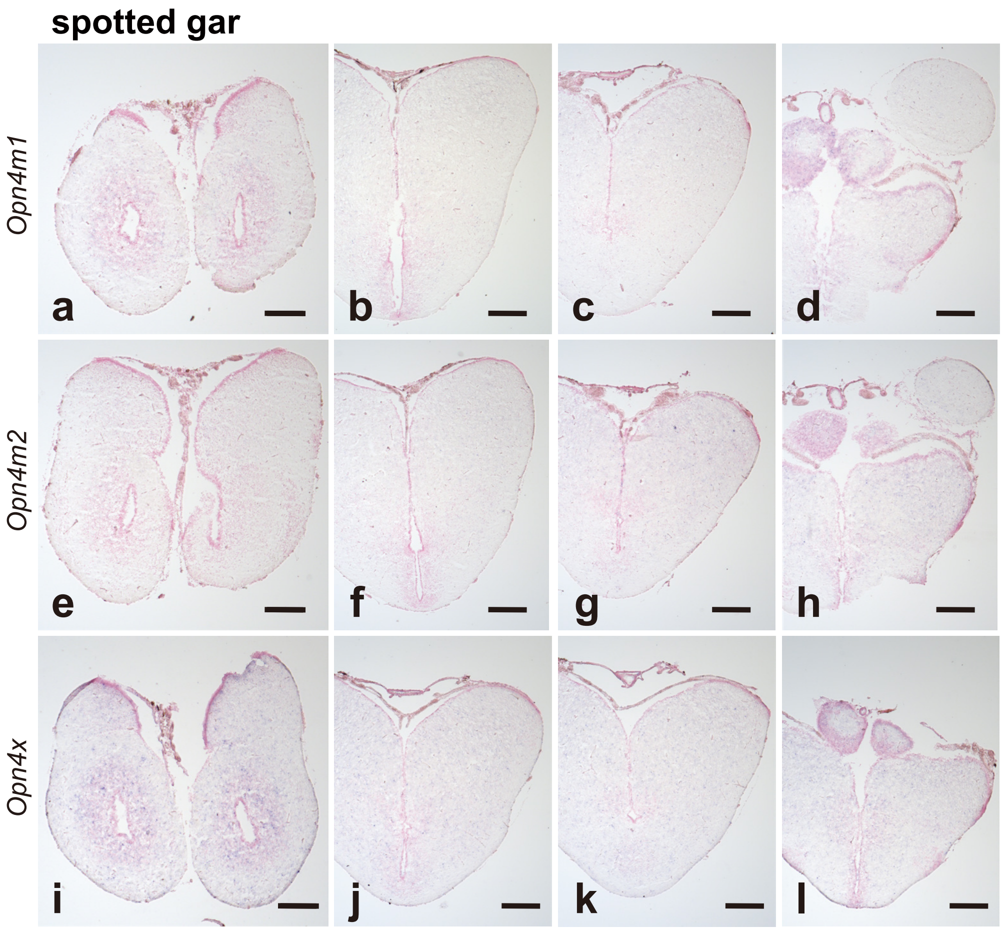

Supplementary Figure 3 Distribution of *Opn4* mRNA in the brain of spotted gar
The transcripts of spotted gar *Opn4m1* (**a-d**), *Opn4m2* (**e-h**) and *Opn4x* (**i-l**) were hybridized with sense probes in the coronal sections of the brain. The sections which were hybridized with the corresponding antisense probes are shown in Fig. 8. Scale bar: 200 μm.

Supplementary Figure 4

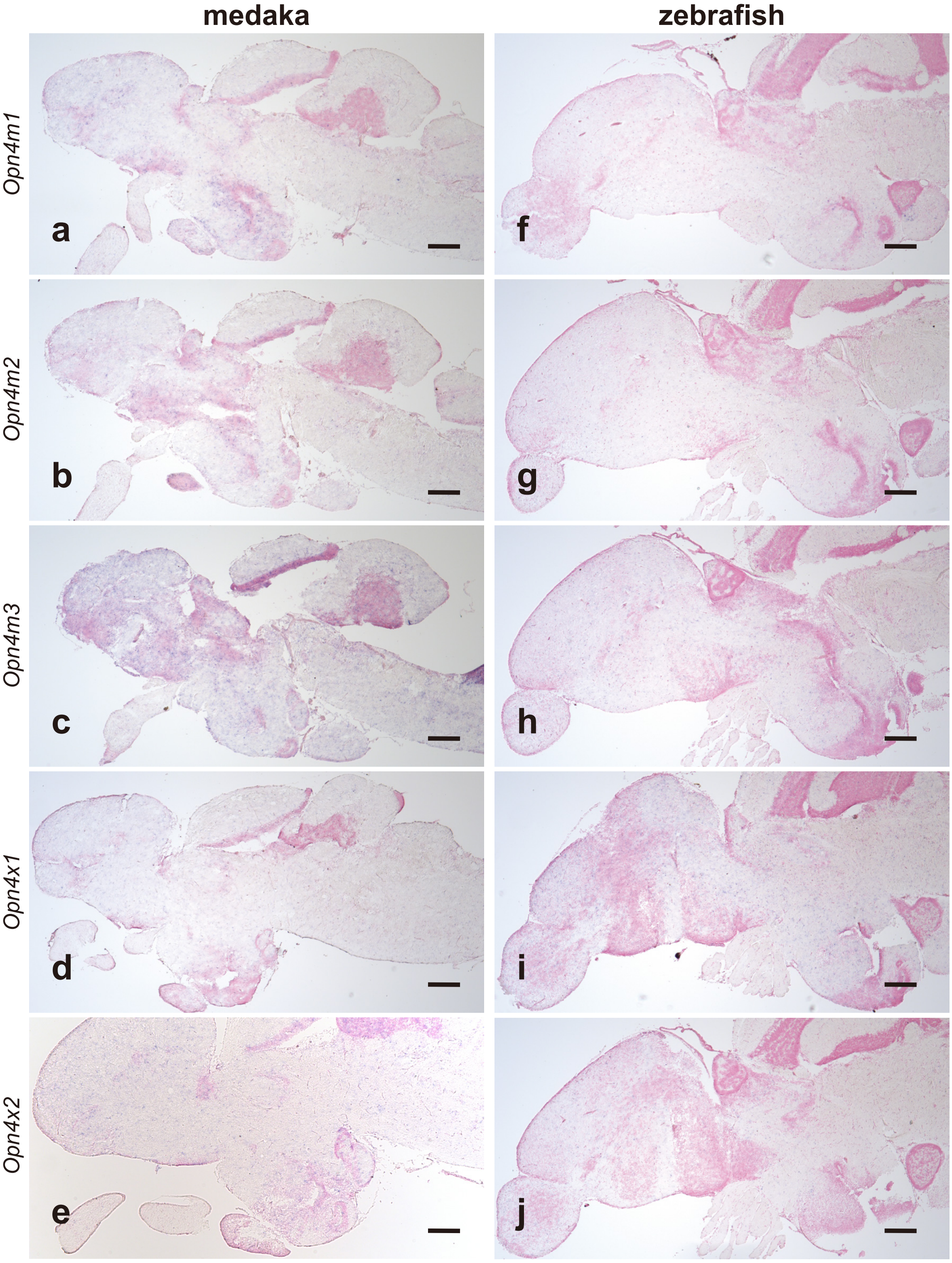

Supplementary Figure 4 Distribution of *Opn4* mRNA in the brain of medaka and zebrafish
a-e The transcripts of medaka *Opn4m1* (**a**), *Opn4m2* (**b**), *Opn4m3* (**c**), *Opn4x1* (**d**) and *Opn4x2* (**e**) were hybridized with sense probes in sagittal sections of the brain. **f-j** The transcripts of zebrafish *Opn4m1* (**f**), *Opn4m2* (**g**), *Opn4m3* (**h**), *Opn4x1* (**i**) and *Opn4x2* (**j**) were hybridized with sense probes in sagittal sections of the brain. The sections which were hybridized with the corresponding antisense probes are shown in Fig. 9. Scale bar: 200 μm.

Supplementary Figure 5

	181
bovive rhodopsin	WVMALACAAPPLVGWSRYIPEGMQCSCGID
human LWS	WIWAAVWTAPPIFGWSRYWPHGLKTSCGPD
human MWS	WIWAAVWTAPPIFGWSRYWPHGLKTSCGPD
mouse MWS	WVWAAIWTAPPIFGWSRYWPYGLKTSCGPD
rabbit MWS	WIWAAVWTAPPIFGWSRYWPYGLKTSCGPD
spotted gar LWS	WVWSAGWCAPPVFGWSRYWPHGLKTSCGPD
zebrafish LWS-1	WVWAAAWCAPPIFGWSRYWPHGLKTSCGPD
zebrafish LWS-2	WVWAAVWCAPPIFGWSRYWPHGLKTSCGPD
guppy LWS-1	WVWAAVWCAPPIFGWSRYWPHGLKTSCGPD
guppy LWS-2	WVWPAVWCAPPIFGWSRYWPHGLKTSCGPD
guppy LWS-3	WVWSAAWCAPPIFGWSRFWPHGLKTSCGPD
guppy LWS-4	WVWSAVWCAPPVFGWSRYWPHGLKTSCGPD
sheepshead minnow LWS-1	WVWSAVWCAPPIFGWSRYWPHGLKTSCGPD
sheepshead minnow LWS-2	WVWPAVWCAPPIFGWSRYWPHGLKTSCGPD
sheepshead minnow LWS-3	WVWSAVWCAPPVFGWSRYWPHGLKTSCGPD
sheepshead minnow LWS-4	WVWSVVWCAPPLFGWSRFWPYGLKTSCGPD

Supplementary Figure 5 Comparison of the amino acid residues among vertebrate long wavelength sensitive cone visual pigments

Most long wavelength sensitive cone pigments (LWS/MWS opsins) in vertebrates have a conserved histidine residue at position 181 (based on the bovine rhodopsin numbering system), which contributes to the binding of Cl^- to induce the red-shift of λmax (37). By contrast, the opsins of the species in the Glires, such as mouse (*Mus musculus*) in the Rodentia and rabbit (*Oryctolagus cuniculus*) in the Lagomorpha, have a tyrosine resides at the corresponding position, which is responsible for their blue-shifted λmax (38-40). Among the LWS retrogenes (*S180r*) in the Cyprinodontiformes, guppy (*Poecilia reticulate*) LWS-4 in the Poeciliidae has a histidine residue at position 181, whereas sheepshead minnow (*Cyprinodon variegatus*) LWS-4 in the Cyprinodontidae has a tyrosine residue at this position. Thus, it can be speculated that H181Y mutation in the red cone pigments occurred independently in the Glires and in the Cyprinodontidae. NCBI accession numbers are as follows: bovine rhodopsin, AB062417; human LWS, NM_020061.6; human MWS, NM_000513.2; mouse MWS, AF011389.1; rabbit MWS, NM_001322264.1; spotted gar LWS, XM_006625291.2; zebrafish LWS-1, AF109371.1; zebrafish LWS-2, BC076120.1; guppy LWS-1, AB748984.1; guppy LWS-2, AB748986.1; guppy LWS-3, AB748987.1; guppy LWS-4, AB748988.1; sheepshead minnow LWS-1, XM_015398975.1; sheepshead minnow LWS-2, XM_015399028.1; sheepshead minnow LWS-3, XM_015398985.1; sheepshead minnow LWS-4, XM_015369304.1.

Supplementary References

37. Wang, Z., Asenjo, A. B., and Oprian, D. D. (1993) Identification of the Cl(-)-binding site in the human red and green color vision pigments. *Biochemistry* **32**, 2125-2130
38. Sun, H., Macke, J. P., and Nathans, J. (1997) Mechanisms of spectral tuning in the mouse green cone pigment. *Proc Natl Acad Sci U S A* **94**, 8860-8865
39. Davies, W. I., Wilkie, S. E., Cowing, J. A., Hankins, M. W., and Hunt, D. M. (2012) Anion sensitivity and spectral tuning of middle- and long-wavelength-sensitive (MWS/LWS) visual pigments. *Cell Mol Life Sci* **69**, 2455-2464
40. Yamashita, T., Nakamura, S., Tsutsui, K., Morizumi, T., and Shichida, Y.(2013) Chloride-dependent spectral tuning mechanism of L-group cone visual pigments. *Biochemistry* **52**, 1192-1197
